# On the Possibility of Designing an Advanced Sensor with Particle Sizing Using Dynamic Light Scattering Time Series Spectral Entropy and Artificial Neural Network

**DOI:** 10.3390/s22103871

**Published:** 2022-05-19

**Authors:** Dan Chicea, Aleksandr S. Doroshkevich, Andriy Lyubchyk

**Affiliations:** 1 Research Center for Complex Physical Systems, Faculty of Sciences, Lucian Blaga University of Sibiu, 550024 Sibiu, Romania; 2 Donetsk Institute for Physics and Engineering Named After O.O. Galkin, NAS of Ukraine, 46 Prospect Nauky, 03028 Kyiv, Ukraine; doroskevich1977@gmail.com; 3 Nanotechcenter LLC, Krzhizhanovsky Str. 3, 03680 Kyiv, Ukraine; se.lyubchyk@fct.unl.pt; 4 REQUIMTE, Faculdade de Ciências e Tecnologia, Universidade Nova de Lisboa, Quina de Torre, 2829-516 Caparica, Portugal

**Keywords:** Dynamic Light Scattering, time series, spectral entropy, Artificial Neural Network, particle sizing, advanced sensor

## Abstract

Dynamic Light Scattering is a well-established technique used in particle sizing. An alternative procedure for Dynamic Light Scattering time series processing based on spectral entropy computation and Artificial Neural Networks is described. An error analysis of the proposed method was carried out and the results on both the simulated and on the experimental DLS time series are presented in detail. The results reveal the possibility of designing an advanced sensor capable of detecting particles with a size bigger than a threshold using this alternative for processing the DLS time series.

## 1. Introduction

Dynamic Light Scattering (DLS) is a technique used for sizing particles in liquid suspensions, typically in water. It was established quite some time ago [1,2] and was initially named Photon Correlation Spectroscopy (PCS) [3,4].

The DLS technique requires a coherent monochromatic light source that has as its target the particles in suspension. Each particle in the beam area produces a quasi-elastic scattering of the incident beam. The wavelets emitted by all of the particles interfere in each location of the space. If a detector is placed in the interference field the scattered light intensity is transformed into an electric potential difference, proportional to the intensity. This electric potential difference can be recorded at equal time intervals producing a DLS time series (TS) that can be processed later.

The particles undergo a continuous chaotic motion, traditionally called Brownian motion, and therefore both the intensity and the phase of the wavelets constantly and randomly change in time [5,6]. The interference field carries information regarding all the particles in the beam area, therefore the DLS TS can possibly be analyzed to provide information regarding the size of the particles in suspension, which is the essence of the DLS technique.

DLS TS can be processed in a simplified manner, to produce the average diameter of the suspended particles, as in [7,8,9,10], or to estimate the particle size distribution, following different assumptions and approximations, as Maximum Entropy algorithms [11,12] or CONTIN [13,14]. The maximum entropy method [11,12] is an advanced method based on fitting. An entropy value is assigned to each solution and the one with the maximum entropy is chosen to be the solution. The fitting procedure in the maximum entropy method is computationally intensive.

Another frequently used procedure for assessing the particle size distribution is CONTIN, which uses the inverse Laplace transform [13,14]. The inverse Laplace transform is sensitive to noise when applied to numerical data, therefore filtering is required [15], which is a computation-intensive procedure [16]. Moreover, because the inverse Laplace transform is an ill-posed mathematical problem, the numerical procedure might lead to ambiguous results. The CONTIN procedure deals with this aspect by introducing a regularization that considerably reduces the number of structural items in expressions. The parameter that is introduced has a big influence on the resulting solution. Choosing the proper value of this parameter can be a more or less educated guess, and can lead to incorrect solutions if the choice is inadequate.

There are other procedures that have been used in processing experimentally recorded data, based on Artificial Neural Networks (ANN) [17,18], DLS TS being included. An averaged scattered light intensity frequency spectrum was used as the input in the work reported in [19]. The ANN had three layers and had the average diameter of the suspended particles as output. The size range was up to 350 nm, very small though, but the work was a proof of concept for using ANNs to process DLS TSs. A continuation of the work in [19] is reported in [20] and uses the autocorrelation of the DLS TS as input to an ANN. The output was the average diameter of the particles, as well, but the range of the particle size was increased to 1200 nm. Both ANNs reported in [19,20] proved to be several thousand times faster than fitting either the Lorentzian line to the frequency spectrum [19] or the autocorrelation [20], with small relative errors. An improvement in the use of ANNs is presented in [21], where the range of the particle size to be measured was extended up to 6000 nm.

Different nonlinear dynamical methods have been used in the last few decades for extracting meaningful information from physiological TS. Among them, Information entropy type TS analysis became widely used [22,23,24,25,26,27,28,29,30,31]. In Physics, entropy is a state function of a thermodynamic system which is a measure of the degree of disorder and is related to the possible number of microscopic states in which the system could be arranged for the same macroscopic system state [32]. Information entropy was introduced by Shannon [33,34] and is considered to be a measure of how “surprising” the outcome of a variable can be.

Several flavors of entropy or entropy-like functions have been defined and used in the last few decades. Some of them are the approximate entropy [22,23,35], permutation entropy [29,36], sample entropy [37], directional entropy [38], and transfer entropy [39].

Information entropy is used as a tool for TS analysis in this work, as an unconventional manner of looking at DLS time series. The next section will describe the DLS particle sizing procedure, considered as a reference, the procedure used in computing the TS entropy, and the algorithm that was used to generate the simulated time series. The correlation that was found between the particle size and the entropy is discussed in detail, together with error analysis and a possible application of the results in designing an advanced sensor capable of estimating the size of the suspended particles using the TS entropy and an ANN to predict the average size of the particles in suspension from the TS computed entropy.

## 2. Materials and Methods

### 2.1. Spectral Entropy Calculation

The entropy that has been computed for TS and is reported in this work is a type of information entropy (Shannon entropy), [33,34], an approximate entropy, more precisely, a spectral entropy (SE). The SE of a signal can be viewed as a measure of the spectral power distribution. The TS is projected to the frequency domain using a Fourier transform. As the TS is a succession of values, the Fast Fourier Transform (FFT) algorithm was used [40,41]. If x(t) is the TS, FS is the frequency spectrum of the TS, obtained from the TS using the FFT procedure. The power spectrum *S(n)* is:

(1) $$S\left(n\right)={\left|FS\left(n\right)\right|}^{2}$$

The SE considers the *S*(*n*) as a probability distribution and calculates the Shannon entropy. The probability distribution is, therefore:

(2) $$P\left(n\right)=\frac{S\left(n\right)}{\sum_{i=1}^{N}S\left(i\right)}P$$

and the spectral entropy SE is [42]:

(3) $$\mathrm{SE}=-\overset{N}{\underset{i=1}{\sum }}P\left(i\right)\log_{2}P\left(i\right)\;$$

where *N* is the number of frequency points.

The normalized spectral entropy SEN is defined as:

(4) $$\mathrm{SEN}=\frac{\mathrm{SE}}{\log_{2}\left(N\right)}$$

In Equation (4) the denominator represents the spectral entropy of white noise [42]. At this point, it is worth mentioning that the SE can be computed for a window of the TS or for the whole TS. In this work, the TSs are DLS TSs, simulated or recorded for stable samples (at least for the time span of a recording, which is several seconds), therefore the SE is computed for the whole TS.

Moreover, the abovementioned algorithm implemented in MATLAB 2021 function *pentropy* was used for the calculations and the results are presented in this work.

### 2.2. DLS Particle Sizing Algorithm

DLS is a quite well-established technique used in particle sizing and it has been constantly developed and improved over the last few decades [2,43,44,45].

**Figure 1:**
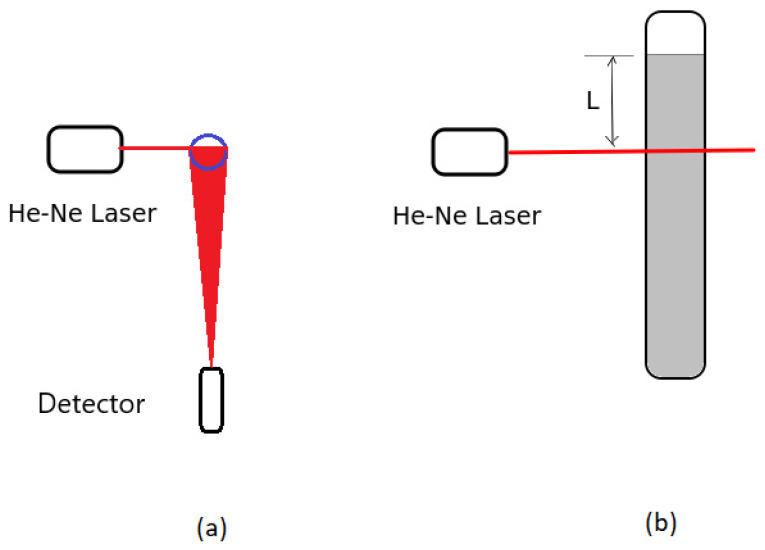
(**a**) The DLS setup, view from above; (**b**) The sedimentation setup.

The setup is quite simple and consists of a coherent light source, which can even be a laser diode, and a transparent container with particles in suspension. The setup also includes a detector that converts the light intensity into an electric signal and a data acquisition system (DAS) that transforms the electric signal into a digital TS, which is recorded and processed later. The simple DLS setup has been presented in recently published articles such as [7,8,19,20,21,46,47,48], some of them published under open access, therefore a schematic only is presented in Figure 1a.

The DAS sampling rate was 16 kHz both for the simulated and for the experimentally recorded TS and the scattering angle to do the recording was chosen to be 90°. Data acquisition was carried out using a conventional DAS system [49]. The cuvette—detector distance was 0.1 m and the glass cuvette inner diameter was 0.01 m. The scattering angle θ was 90°, temperature was 20 °C, and the dynamic viscosity coefficient η was 0.001 kg·m/s.

The data recorded by the detection chain consisting of a photodetector, amplifier and DAS, at equally spaced time intervals, is the TS, which is the succession of values:

(5) $$x_{1},x_{2},x_{3},\ldots ,x_{N}$$

and the normalized autocorrelation ACR of the TS, whether experimentally recorded or simulated, is:

(6) $$ACR\left(\tau \right)=\frac{\left\langle x\left(t\right)|x\left(t+\tau \right)\right\rangle }{\langle x{\left(t\right)\rangle }^{2}}\;$$

In (8) n is the refractive index of the solvent, ***λ*** is the wavelength of the coherent incident light in vacuum, and ***θ*** is the scattering angle. The work presented here was carried out with the purpose of designing an advanced sensor, not to improve the precision of the DLS method, therefore the approximation that the particles can be considered to have a mono-sized distribution was used. Within this approximation, the autocorrelation ACR can be written as [1,2,44]:

(7) ACR(τ)=1+exp(−2qDτ)

where *q* is the scattering vector detailed in Equation (8), ***β*** is a coefficient that depends on the experimental configuration and can be adjusted to be 1, as was executed in the work reported here:

(8) $$q\left(\theta \right)=\frac{4\pi n}{\lambda }\sin\frac{\theta }{2}$$

In (7) *D* is the diffusion coefficient, which depends on the average diameter *d* of the suspended particles as in Equation (9), the Stokes–Einstein equation [50]:

(9) $$D=\frac{k_{B}T}{3\pi \eta d}$$

In (9) *k_B_* is Boltzmann’s constant, *T* is the absolute temperature of the suspension, *η* is the dynamic viscosity coefficient of the solvent.

The DLS procedure that was considered as a reference for the work presented here computed the ACR of the TS, as in Equation (6). The function described by Equation (7) with *β* = 1 and 1 subtracted from it is the normalized ACR. Fitting the normalized ACR to the ACR computed on experimental data with Equation (6) using a nonlinear least-squares minimization procedure to determine the best match for D, and therefrom the most probable diameter *d* by reverting Equation (9) was considered to be the reference procedure for DLS TSs processing.

An alternative to Equation (7) for describing the normalized ACR decay would consist of using a combination of two exponentials, under the assumption that the sample consists of two types of mono-sized particles. This alternative was not used though, for two reasons. Firstly, the work presented here is on the line of simplifying the experimental setup and the data processing procedure for DLS. Using the fit of a combination of two or more exponentials to describe the normalized ACR is not within the remit of this work. Secondly, a combination of two exponentials has three parameters to be determined by a minimization procedure, the two diffusion coefficients at the exponent, and one multiplication coefficient. Such a fit, with three free parameters, is not fully reproducible and the result might depend on the start values chosen for the parameters, a choice that can be more or less educated. While we can chose start values close to the real ones for a known sample, such a choice can be difficult for an unknown sample and can lead to a local minimum, therefore, to values that are relatively far from the accurate values. With these in mind, the ACR was described using Equation (7), with the experimental setup in such a manner that *β* = 1 (average speckle size equal with the size of the detector) and 1 subtracted to have an exponential decay of the values computed from the experimental TS.

In order to estimate the relative errors in assessing the diameter of the suspended particles using the procedure described above, we understand that the argument of the exponential in Equation (7), y, as described in Equation (10) is actually assessed. If we replace q from Equation (7) and *D* from Equation (9) we find for *y*:

(10) $$y=2Dq=\frac{8k_{B}n}{3\eta \lambda }\cdot \frac{T}{d_{DLS}}\cdot \sin\frac{\theta }{2}\;$$

In Equation (10) *d_DLS_* is the diameter assessed by a least-squares minimization procedure, and from (10) we find the diameter *d_DLS_*:

(11) $$d_{DLS}=\frac{8k_{B}n}{3\eta \lambda }\cdot \frac{T}{y}\cdot \sin\frac{\theta }{2}\;$$

The constant quantities were grouped in the first factor. If we differentiate the logarithm of $d_{DLS}$ we find:

(12) $$\frac{d\;\left[d_{DLS}\right]}{\left[d_{DLS}\right]}=\frac{dT}{T}+\frac{dy}{y}+\frac{1}{2}\frac{d\theta }{\tan\frac{\theta }{2}}\;$$

Taking the worst scenario when all the errors cumulate, and considering the differential of the physical quantities to be the errors in measuring them, we find:

(13) $$\varepsilon_{\left[d_{DLS}\right]}=\frac{\Delta \;\left[d_{DLS}\right]}{\left[d_{DLS}\right]}=\frac{dT}{T}+\frac{dy}{y}+\frac{1}{2}\frac{d\theta }{\tan\frac{\theta }{2}}$$

If we consider Δ*T* to be 1 K and *T* = 293.15 K (20 °C), a generic 0.03 = 3% relative error for the least-squares fit on y and 3 degrees (expressed in radians in Equation (13)) for Δ*θ* for the scattering angle *θ* of 90 degrees we find for $\varepsilon_{d_{DLS}}$ a value of 0.06 = 6%, and this will be used later in the work as the relative error in assessing the DLS diameter, and in plotting the error bars, as well.

It is worth mentioning here that a source of systematic errors might be the polydispersity of the sample, not taken into account here. Reference [6] reveals that when bigger particles are present in suspension, the intensity of the light scattered by these particles is considerably bigger than the light scattered by small particles, therefore the interference field is overwhelmingly dominated by big particles. Therefore the $d_{DLS}$ that we find by analyzing the DLS TS will have as its output an average of the diameter of the bigger particles.

Another source of systematic errors lies in the fact that DLS outputs the hydrodynamic diameter, slightly bigger than the physical diameter [8]. The AFM technique, though, can output the physical diameter, with certain precautions regarding the size of the nanoparticles and the radius of the tip [51,52], which can be bigger than the nanoparticle and can increase during scanning, as it wears out.

### 2.3. DLS TS Generation

In order to establish and verify a possible correlation between the TS SEN and the diameter of the suspended particles that produces the scattered light time series, a large set of TS and the corresponding diameter are required when using ANNs. Latex balls with well-known diameters are commonly used for particle sizing devices’ calibration, but the diameters come in big diameter steps rather than continuous, and they have a certain diameter size distribution around the indicated value.

Simulated TS appear to be a reasonable alternative, as they can be generated for suspended particles with diameters increasing with a small step and over an extended size range. Such simulated TS have successfully been used to train ANNs for processing DLS time series and the results are reported in papers such as [19,20,21]. As the algorithm was described in detail, it will be presented in this section briefly.

The FFT procedure applied to a TS views the signal as a sum of harmonic functions, each having an amplitude and a phase. The FFT produces the amplitudes and the phases of each frequency component and the collection is called a frequency spectrum of the signal. If the signal is a DLS TS, the scattered light intensity spectrum can be described by the Lorentzian function [2,9,19,20,21]:

(14) $$S\left(f\right)=a_{0}\frac{a_{1}}{{\left(2\pi f\right)}^{2}+a_{1}^{2}}$$

In (14) *a*_0_ parameter does the scaling of the spectrum while *a*_1_ depends on the diameter of the SCs, as described by Equation (15) [19,20,21] and establishes a turnover point of the line in a log–log plot of S vs. f, the frequency:

(15) $$d=\frac{2k_{B}Tq^{2}}{3\pi \eta a_{1}}\;$$

The signal can be composed back as a sum of harmonic functions having a discrete set of frequencies and an amplitude corresponding to each frequency, and this procedure was used to generate the simulated DLS TSs. A discussion and analysis on the number of frequencies required to produce a realistic DLS TS is presented in detail in [20], and the conclusion was also used in the TSs generated for this work. For a total number of N data points in a TS, a number of frequencies *Nf* = *N*/2 + 1 equally distributed on the frequency spectrum were therefore used.

The TS indicated as *x(t)* was generated at equal time intervals, such as having been recorded with a data acquisition rate of 16 kHz, as:

(16) $$x\left(t\right)=\overset{Nf}{\underset{j=1}{\sum }}A\left(f_{j}\right)\cdot \cos\left(2\pi f_{j}t+\varphi_{j}\right)\;$$

where *A*(*f_j_*), the amplitude of the *j*-th component, was computed as the square root of *S*(*f_j_*) in Equation (16) [19,20,21]. In Equation (16), sine can be used instead of cosine, as reported in [19,20,21] with no significant difference, as the initial phases *φ_j_* were generated randomly in the interval [0–2π].

In order to simulate the DLS TS in a realistic manner, an amount of noise was added. Reference [21] describes a procedure that can be used to add both a white-type of noise *x_noise_* and a 50 Hz component and the harmonics, *x_h_*, as the noise of the power grid is ubiquitarian. The noise was generated as a sum of harmonic functions, both with 50 Hz and harmonics as frequencies and with a number of 300 frequencies randomly generated over the expected frequency range of the frequency spectrum. Such a combination of noise contains the sum of harmonic functions, which is completely predictable, therefore causing a decrease of the entropy. In order to simulate noise in a more realistic manner, white noise only was added. The noise TSs were generated separately and added to the TS. First, the amplitude A of the TS, as generated using Equation (16) and the procedure described above, was determined as the difference between the maximum and the minimum of the whole set of data. An amplitude of the added noise, *A_noise_*, was established as a percentage of the A, 1.5% more precisely, and this value was found by successive iterations. A noise TS, *x_noise_* was computed as having the same quantity of data as the TS intended to be altered, each data point consisting of a random number uniformly distributed in the range [*−A_noise_, A_noise_*], as in Equation (17). The *rand* function of the MATLAB 2021b was used, and this function generates random numbers with uniform distribution in the interval [0, 1]. The seed was initiated using the *shuffle* option; therefore, the set was different from each other for each noise TS generated:

(17) $$x_{noise}\left(t\right)=2A_{noise}\left(rand-0.5\right)$$

Finally, the TS was calculated by adding *x_noise_* of Equation (17) to *x* in Equation (16).

### 2.4. Sample Preparation

The particle sizing method based on computing the SEN, and from it the average diameter, was tested both on simulated data and on data from the real world. For this purpose, crystalline Barium sulfate BaSO_4_ (Sigma Aldrich, Darmstadt, Germany, 99%) was chosen for preparing an aqueous suspension, because it is insoluble in water. The density of the crystalline powder is 4.49 g/cm^3^. An amount of 2 g of the crystalline powder was manually milled using a synthetic Sapphire mortar and pestle for 15 min to reduce the crystallite size, in order to produce a powder of a smaller grains’ size, in the range of nanoparticles. A total of 0.1 g of the milled crystallin powder was mixed with 20 cm^3^ of deionized water. A glass circular cuvette of 1 cm diameter was used as a sample container, both for the sedimentation and for the target of the DLS experiment and was sealed after adding the suspension to prevent evaporation and thus a change in the concentration, which might slightly modify the DLS diameter.

Sedimentation as sample preparation has been described in detail in [19], therefore it will be only briefly described in this section.

If a spherical particle of diameter *d* and density *ρ* is submerged in a fluid of density *ρ*_0_ and dynamic viscosity coefficient *η* the vector sum of gravity and buoyant force accelerates it vertically, downwards if *ρ* < *ρ*_0_, such as is the case of the BaSO_4_ particles. As it moves, the drag force in the Stokes regime makes itself manifest, in the opposite direction of the velocity. At equilibrium, when the vector sum of the three forces is null, the steady state velocity *v_l_* is:

(18) $$v_{l}=\frac{\left(\rho -\rho_{0}\right)g}{18\eta }\cdot d^{2}$$

where *g* is gravitational acceleration. This strong variation in the equilibrium falling velocity with the square of the diameter can be used to produce a sample that has a decrease of the maximum diameter of the distribution of the suspended particles in time, with a setup as depicted in Figure 1b.

The bigger particles fall faster than the smaller particles. For a length *L* from the upper surface of the liquid to the beam location, after time Δ*t* from placing the suspension in the cuvette, only the particles with the velocity *v_l_* smaller than *L*/Δ*t*, will remain in the beam area, therefore only the particles having a diameter smaller than *d_max_*, as described by Equation (19):

(19) $$d_{max}=\sqrt{\frac{18\eta }{\left(\rho -\rho_{0}\right)}\cdot \frac{L}{\Delta t}}$$

The DLS experiment was carried on by recording a DLS TS with a 30 min time interval between them, thus having particles with a diameter decreasing from a measurement to the other, suspended in the beam area. Care was taken when selecting length *L*. A smaller *L* will produce a bigger variation of the diameter from one measurement to the other, but the *L* relative variation caused by the capillary ascension of the liquid on the glass tube walls will be bigger from one location in the cuvette to the other. For this reason, a length L of 2 mm was chosen, but cannot be very accurately stated, because of the capillary ascension that was present for a 1 cm diameter cuvette. The relatively small value for *L* was chosen to make evident a variation in the measured diameter for an experiment lasting for tens of hours, rather than weeks. Nevertheless, the procedure described in this section should be viewed not as a precise particle separation procedure, but as a sample preparation procedure that can be used to have a decreasing biggest diameter of the particles suspended in a solvent.

## 3. Results and Discussion

A TS was generated for each diameter in the set, with a number of 32769 frequencies, having 2^16^ = 65,536 data points each, using Equation (16). For each diameter *d*, the parameter *a_1_* was calculated using Equation (15), and the amplitude *A*(*f_j_*) corresponding to the frequency *f_j_* was calculated as the square root of *S*(*f_j_*) using Equation (14) [21]. The scattering angle was 90° and the scattering vector *q* was calculated using Equation (8), where n = 1.333 was used in simulation, which is the refractive index of water considered to be the solvent and *η* = 0.0010 daP was considered, as it is the dynamic viscosity coefficient of water at 20 °C. Each TS was generated in such a manner that it appears to be sampled at fs = 16,000 Hz.

For each TS, noise was generated and added, as described in Section 2.3.

**Figure 2:**
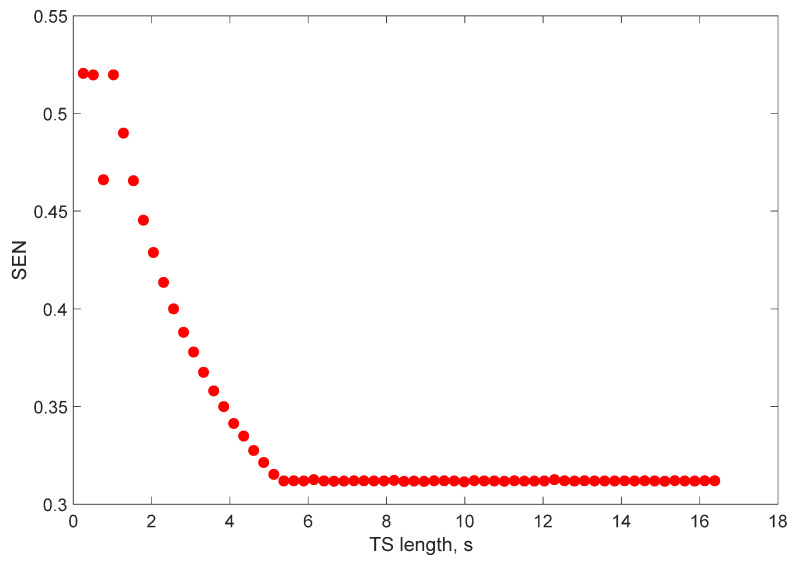
The variation of the SEN with the TS length.

First, a TS was generated for a test diameter of 910 nm. The Shannon normalized entropy SEN was calculated as in Equation (4), using the MATLAB function *pentropy*. The parameter *Scaled* was allowed to be *true*, thus *pentropy* returned the spectral entropy scaled by the spectral entropy of the corresponding white noise [42]. Moreover, the parameter *Instantaneous* was set to *false*, therefore *pentropy* returned the spectral entropy value of the whole signal or spectrum as a scalar [42], not on a slice of the TS. The test TS was used to assess the robustness of the SEN with respect to the TS length. Figure 2 shows the variation of the SEN with the TS length, measured in seconds.

Figure 2 reveals that for short TSs, which is shorter than 5 s, hence containing less than 80,000 data points, the SEN computed as previously mentioned decreases with the length of the TS. Another test was performed on more TSs generated for the same diameter, but containing the same quantity of data, that is 2^16^ = 65,536, and the SEN was found to be the same, within a relative error of 0.5%. With these in mind, the number of data points in each TS was 65,536, for the TSs mentioned in this work.

**Figure 3:**
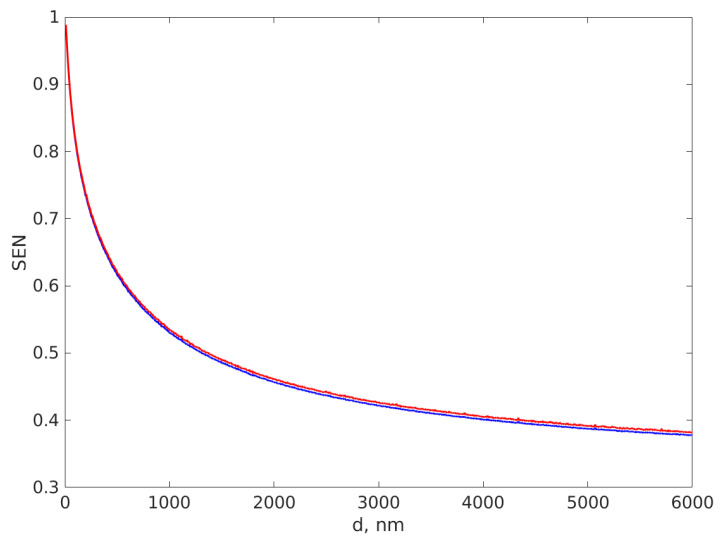
The SEN vs. the diameter used for TS generation. The blue dots stand for the clean TS SEN and the red dots for the noisy TS SEN.

TSs were generated for diameters from 10 nm to 6000 nm, with a step of 5 nm, and were collected in an array. The SEN was computed for each TS, as generated using Equation (16) prior noise addition, clean TS hereafter, and after noise addition as computed using Equation (17), and these are the noisy TSs hereafter. Figure 3 illustrates the SEN vs. the diameter used for TS generation. The blue dots stand for the clean TS SEN and the red dots for the noisy TS SEN. Figure 3 reveals that noise addition increased the computed SEN, and the increase is more significant for the bigger diameter TSs. More importantly, the SEN has a monotone variation with the diameter over the whole diameter range, therefore it is possible to invert the variation to calculate the diameter of the TS from the SEN value. Several functions have been assessed as candidates, but the best result in inverting the variation of the SEN with the diameter was produced by an ANN for fitting.

**Figure 4:**
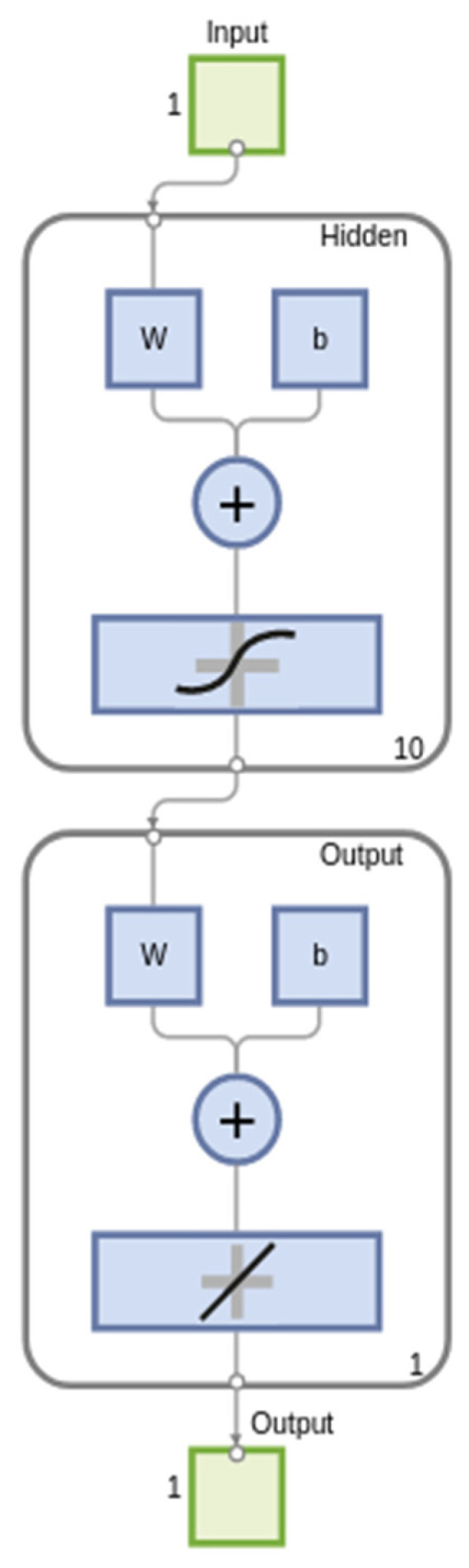
The ANN architecture.

The ANN architecture is presented in Figure 4. It was implemented in MATLAB 2021b and has one input layer with one neuron, one hidden layer with ten neurons, and one output layer with one neuron.

The transfer function for the hidden layer was *tansig* and for the output layer was *linear*. The SEN collection were the input data and the diameters were the targets. The Levenberg–Marquardt algorithm was used for minimization, the sum of the least-squares was optimized, 70% of the set was used for training, 15% of the set was used for validation, and 15% for testing. The overall value for R was 0.99972, which indicates a very good performance of the ANN.

**Figure 5:**
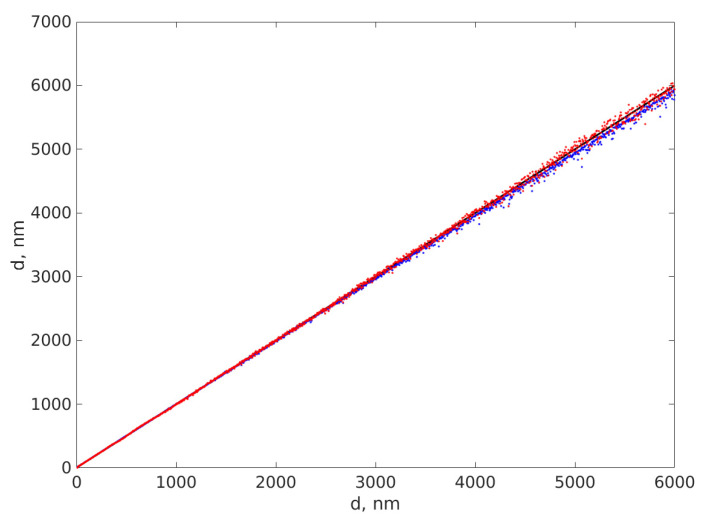
DLS diameters (blue dots); the SEN diameters (red dots); generated diameters (black continuous line); versus the generated diameters.

The diameter of each TS was assessed using the procedure described in Section 2.2, by fitting the expected ACR in Equation (7) to the computed ACR of each TS to assess the diffusion coefficient *D*, and, from here onwards, the diameters of the suspended particles using Equation (9), which we name the DLS diameters. The computed SEN values were reverted using the trained ANN, and produced the diameters that we name hereafter the SEN diameters. Figure 5 depicts the DLS diameters with blue dots, the SEN diameters with red dots, and the diameters used in generating the TSs, which we name the generated diameters, with a black continuous line, versus the generated diameters.

Figure 5 reveals a slight scatter of the diameters around the generated diameters. A distinct perspective on the accurateness of the prediction is given by the absolute errors, Δ*d*, and by the relative errors *εd*, as defined in Equation (20). The diameters *d_SEN_* were computed using the ANN and, as expected, were slightly scattered around the straight line *d* = *d*. As these kinds of data cover more than two orders of magnitude, a better perspective might be offered by a plot of the errors of this novel procedure of assessing the diameters:

(20) $$\Delta d=d_{SEN}-d_{gen};\;\;\varepsilon_{d}=\frac{d_{SEN}-d_{gen}}{d_{gen}}\cdot 100,\;\%$$

**Figure 6:**
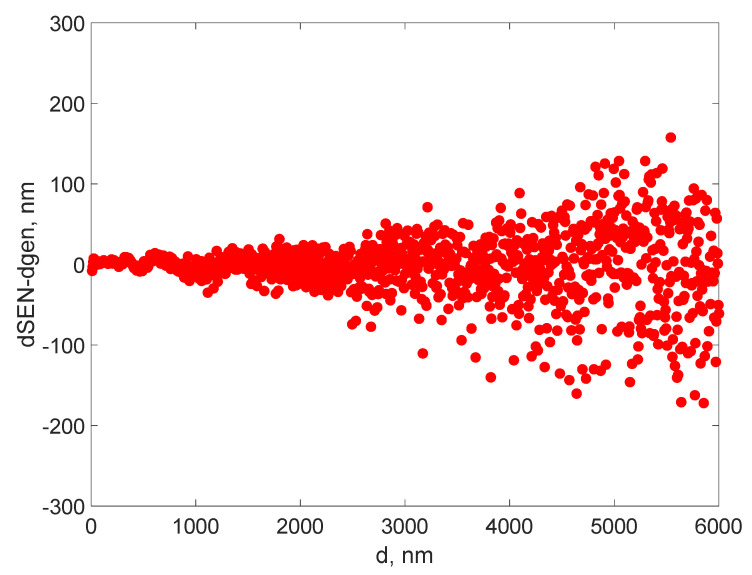
The absolute errors in assessing the *d_SEN_* diameters.

**Figure 7:**
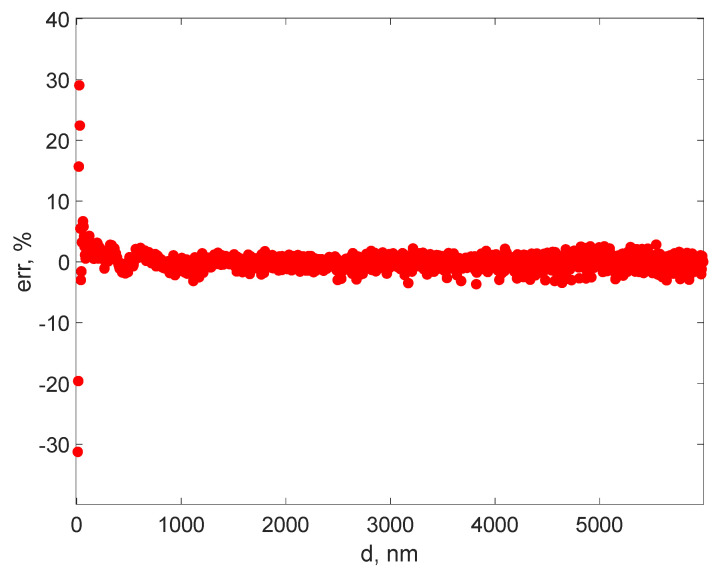
The relative errors in assessing the *d_SEN_* diameters.

Figure 6 and Figure 7 present the absolute errors and the relative errors calculated using Equation (20).

Figure 6 reveals that the absolute errors are scattered around 0, rather than presenting a smooth variation, a trend, or a pattern, indicating that the ANN predicts diameters relatively accurately. The relative errors are quite big in the very small diameter range, which is below 40 nm, and the precision gets better for diameters bigger than 40 nm, and does not exceed the interval [–6, 6]%, as illustrated by Figure 7. This result appears to be encouraging in suggesting the procedure as an approximate method for assessing the average diameter of the suspended particles from the recorded DLS TS. Moreover, the procedure might be used for designing an advanced sensor capable of sensing the presence of particles bigger than a selected triggering value.

**Figure 8:**
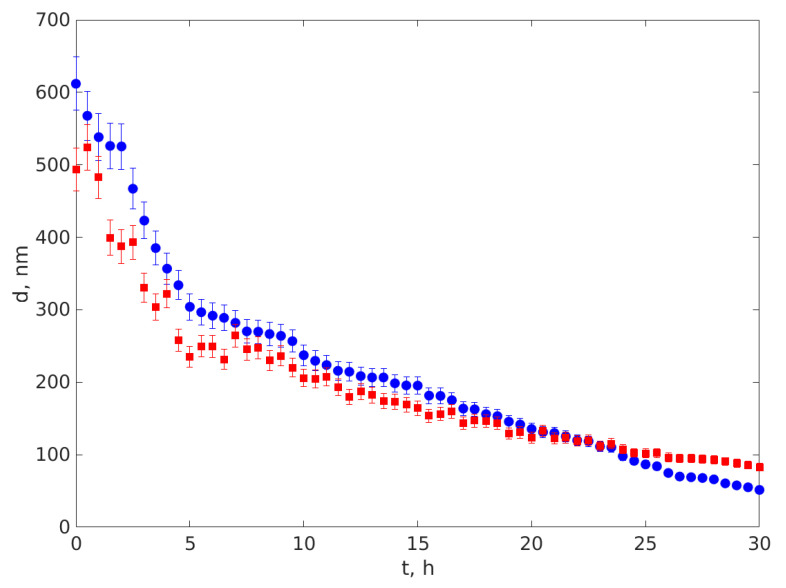
The computed diameters for the sedimentation experiment. The blue dots are the DLS diameters and the red squares are the SEN diameters.

The procedure for assessing the diameter of the suspended particles by recording a DLS TS, computing the SEN, and finding the diameter using a trained ANN was also tested on experimentally recorded TSs. The sample preparation experiment using sedimentation, as described in Section 2.4, was employed in producing the BaSO_4_ suspension. The experiment lasted for 30 h, and a TS was recorded every 30 min. The recorded TSs were processed using both the DLS procedure described in Section 2.1 as reference, producing the diameters named *d_DLS_*, and the procedure described above, based on computing SEN from the diameter using the ANN, having as the output the diameters named *d_SEN_*. The diameters computed as such are illustrated in Figure 8. The error bars were calculated as 6% of the computed value, as described in Section 2.2, both for *d_DLS_* and for *d_SEN_*.

Figure 8 reveals that the diameters computed using the two procedures, the reference DLS and the newly described SEN-based procedure, indicate the same correct decreasing trend for the diameters that remained in the coherent beam area as the sedimentation continued. The differences between the computed diameters are bigger than the error bars, which can be explained considering the systematic error caused by the assumption of having monodispersed particles in suspension. Another explanation lies in the data used to train the ANN, which is based on entropy. Entropy is an additive parameter and adding random noise increased the entropy. Figure 5 illustrated that greater entropy corresponds to smaller diameter of particles in the samples. Equation (17) describes the noise added to the simulated TSs to mimic the experimentally recorded TSs. The adding noise procedure requires improvement, as it appears to add less noise than is optimal on the TSs corresponding to the smaller particles, and more than is optimal for the bigger particles. Improving this noise addition algorithm is scheduled for improvement. Nevertheless, despite the differences that are slightly bigger than the estimated error bars, the two curves are very close, and the procedure can be used in designing a sensor capable of detecting particles bigger than a threshold.

**Figure 9:**
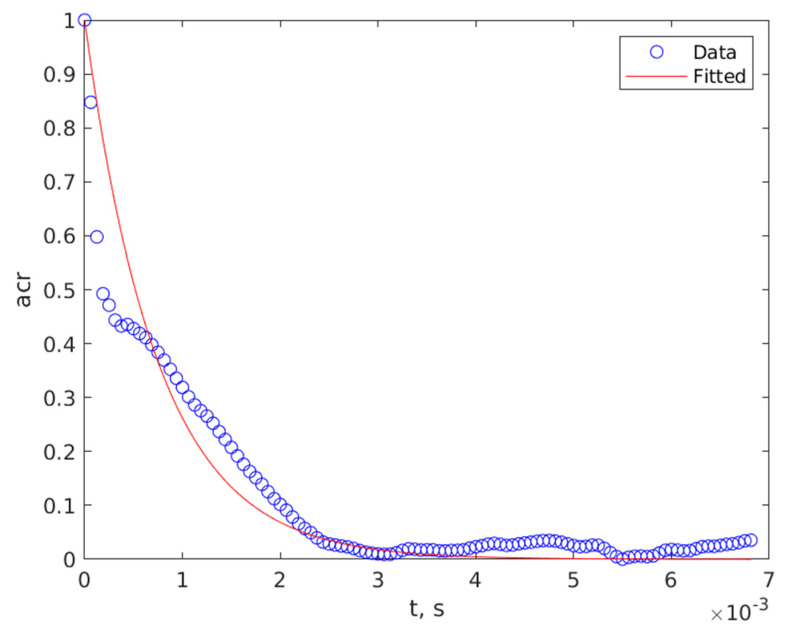
The ACR of a recorded TS, blue circles; and the best fit, continuous red line.

Figure 9 reveals that for the first lags the ACR computed on the experimental TS has a faster decrease than the fit line followed by a slower decrease, which suggest that the sample is not monodispersed but contains particles with different sizes, which is quite normal considering the milling procedure prior to dissolving the crystalline BaSO_4_ sample. Nevertheless, the approximation appears to be reasonable and the DLS procedure outputs an approximate diameter of the particles suspended in solvent and explains the differences in the diameters computed using the DLS and the SEN procedure.

As previously described and clearly highlighted and presented in [6], the interference landscape is dominated by light scattered by the bigger particles, but smaller particles have a contribution to make, as well. This is clearly illustrated in Figure 9 that depicts the ACR of a recorded TS and the best fit. The output of the reference procedure is an average of the bigger diameters in the sample. As the procedure described in this paper is intended to be used in designing a sensor capable of detecting particles bigger than a threshold, the procedure described as a reference in Section 2.2 appears to be appropriate.

## 4. Conclusions

DLS, which is a well-established technique used in particle sizing, was revisited with respect to the TS processing. The distribution of the suspended particles was approximated to be mono-dispersal or mono-dispersal-like. The reference DLS TS processing method used a least-squares minimization procedure to fit the exponentially decreasing expected normalized ACR to the computed ACR, to assess the diffusion coefficient and from here, the average diameter of the suspended particles. The same TSs were processed in a novel manner, which involved computing the normalized spectral entropy SEN of the whole TS, having exactly 2^16^ = 65,536 data points. The novelty brought by this work consists of revealing the monotonous correlation between the average diameter of the suspended particles and the SEN that was found to exist. An ANN was trained to output the average diameter, having the SEN of the TS as input. Training was carried on using a big set of simulated TSs with white noise added to them.

The procedure was tested on experimentally recorded data, as well, during a sedimentation experiment where the diameter of the particles in the beam area continuously decreased. The errors, as compared with the reference DLS, were found to be slightly bigger than the theoretically estimated errors for the particles at the beginning of the sedimentation experiment, in part because the range of particles in suspension at the beginning of the experiment was bigger than at the end. Nevertheless, the novel procedure described here can be used in assessing the particle size of the particles in suspension. In order to increase the precision, a DSL TS can be recorded on the same experimental setup on pure solvent and used in establishing a better approach for the noise to be added on the set of simulated TSs used in training the ANN.

As mentioned in the title of this paper, the whole procedure can be viewed as a proof of concept, as steps towards designing an advanced sensor capable of detecting particles bigger than a threshold. In order to design a sensor, experimental TSs recorded on many suspensions containing particles with well-known mono-sized particles can be used to calculate the collection of SEN that are used to train the ANN. In this alternative, which uses experimental TSs recorded on the same experimental setup as the sensor to train the ANN, differences as in Figure 8 would not be present any longer.

The procedure described here did not appear to be more precise than the reference DLS procedure; it represents an alternative for processing DLS TSs, based on computing the SEN and reverting it to assess the diameter of the particles. The amount of computation is less than for fitting a function to the data by the least-squares minimization procedure. It is hard to state a precise value for how much less computation (expressed in floating point operations or in computing time) is required, as fitting strongly depends on the start parameters and on the precision that is indicated, which can be bigger than the actual required precision. However, using an ANN for computing is much faster, as is reported in [19,20,21], therefore the novel procedure reported here can possibly be migrated onto a light computation platform and might be integrated and used for designing a sensor that is sensitive to the size of the particles in suspension, such as fluids for biological or health applications, where the presence of bigger particles such as bacteria might be critical.
